# Integrative single-cell transcriptomics and mendelian randomization identifies BTN3A2 as a shared protective factor in Behçet’s disease and inflammatory bowel disease

**DOI:** 10.3389/fimmu.2026.1845143

**Published:** 2026-06-24

**Authors:** Qinjin Dai, Yuelan Wu, Wenbo Geng, Mengna Li, Wanyun Zhang, Yuxian Lai, Pei Zhang, Chunjiang Zhou, Yinuo Wang, Qingfeng Cao, Xiang Luo, Yujie Lai, Changwei Huang, Peizeng Yang

**Affiliations:** 1 Ophthalmology Medical Center, The First Affiliated Hospital of Chongqing Medical University, Chongqing Key Laboratory for the Prevention and Treatment of Major Blinding Eye Diseases, Chongqing Branch (Municipality Division) of National Clinical Research Centre for Ocular Diseases, Chongqing, China; 2 Department of Ophthalmology, Joint Research Laboratory for Ocular Immunology and Retinal Injury Repair, The First Affiliated Hospital of Zhengzhou University, Henan International, Henan Province Eye Hospital, Zhengzhou, China

**Keywords:** Behçet’s disease, inflammatory bowel disease, mendelian randomization, single-cell sequencing, T cell

## Abstract

**Background:**

Behçet’s disease (BD) and inflammatory bowel disease (IBD) are immune-mediated chronic inflammatory disorders. Although they exhibit significant overlap in clinical manifestations and immunological features, whether they share molecular targets and pathogenic links remains unclear. This study aimed to explore the possible linking and shared treatment target between these two diseases.

**Methods:**

We analyzed scRNA-seq data from the peripheral blood of BD and IBD patients to delineate the immune cell landscape and identify key subsets via differential proportion analysis. Combining expression quantitative trait loci (eQTL) and genome-wide association study (GWAS) data, Mendelian Randomization (MR) analysis was performed to infer causal relationships between the key gene expressions in BD and IBD. Finally, validation was performed using the experimental autoimmune uveitis (EAU) mouse model, external datasets, and blood samples from BD uveitis patients.

**Results:**

scRNA-seq analysis revealed a significant decrease in the proportion of CD4^+^ TEM_TH1-like cells in both BD and IBD. We identified 45 key signature genes within this subpopulation, among which the BTN3A2 gene was identified as a protective one in both BD and IBD. Co-localization analysis indicated spatial consistency between the BTN3A2 eQTL and BD GWAS signals. At the cellular functional level, BTN3A2^+^ and BTN3A2^-^ CD4^+^ TEM_TH1-like cells exhibited distinct cell-cell communication networks and pathway enrichment characteristics. The interactions between BTN3A2^+^ and BTN3A2^-^ cells were mediated by distinct signaling pathways in IBD and BD respectively. The EAU mouse model, which recapitulates BD-like ocular inflammation, was found for the first time to display IBD-like colonic pathological, including inflammatory infiltration, crypt dilation, goblet cell loss, and barrier disruption, accompanied by elevated IL-1β expression. Transcriptomic analysis using EAU mouse retina further revealed that the IBD pathway was one of the most significantly enriched disease-related pathways. Independent external datasets showed a significant downregulation of BTN3A2 at mRNA level in IBD, whereas downregulation of BTN3A2 at mRNA and protein levels was found in BD patients with uveitis.

**Conclusions:**

This study identifies BTN3A2 as a shared protective factor in BD and IBD and provides experimental evidence linking BD-associated ocular inflammation to intestinal pathology, establishing a new basis for comorbidity−targeted therapeutic strategies.

## Introduction

Immune-mediated chronic inflammatory diseases pose a significant global health challenge. Characterized by the complex etiology, a long-lasting course and relapsing tissue damage, they lead to a substantial socioeconomic and healthcare burden (1). Among these, Behçet’s disease (BD) and inflammatory bowel disease (IBD) are two prototypical systemic inflammatory conditions that have garnered increasing academic interest due to their significant clinical and immunological similarities.

BD is a multisystem inflammatory disorder rooted in vasculitis, manifesting as recurrent oral ulcers, genital ulcers, uveitis, and skin lesions (2). IBD, which mainly includes Crohn’s disease (CD) and ulcerative colitis (UC), is marked by persistent and recurring inflammation affecting the gastrointestinal tract (3). Despite the differences in primary organ involvement, BD and IBD exhibit a range of shared clinical features. A subset of BD patients presents with intestinal involvement, known as intestinal BD, featuring ileocecal ulcers that closely resemble the phenotype seen in IBD (4). Conversely, IBD patients often exhibit extraintestinal manifestations overlapping with BD, such as oral aphthous ulcers, skin ulcers, uveitis, or arthritis (5). The connection between BD and IBD is further proved at the immunological level. Research indicates that both diseases are characterized by dysregulation of the innate and adaptive immune systems (6–8), where CD4^+^ T cells act as major drivers of the pathogenic inflammatory response (9, 10). In this process, microbial antigens or their mimics processed by antigen-presenting cells can activate the CD4^+^ T cells. This activation shifts the immune balance toward a pro-inflammatory state, which is presented as the activated Th1 and Th17, suppressed regulatory T cells (Treg) function, increased pro-inflammatory cytokines, and promoted neutrophil infiltration in both BD and IBD (11, 12). Furthermore, genetic evidence from genome-wide association studies (GWAS) consolidates this link by pinpointing key shared risk genes for BD and IBD (13). While these findings highlight the commonalities at the pathway-level, a key unresolved issue is whether a shared dysregulated CD4^+^ T cell subtypes and their key regulatory molecules exists in both diseases. Furthermore, the therapeutic strategies for BD and IBD show considerable commonality. The fact that drugs like corticosteroids, sulfasalazine, thalidomide, and tofacitinib are applicable to both conditions suggests the presence of common therapeutic targets (11, 14, 15). Overall, the clinical overlap between BD and IBD implies the shared pathogenic mechanisms that remain to be fully elucidated, while their diagnostic labels stay distinct. Therefore, identification of the common molecular targets is crucial for developing novel targeted therapies.

Traditional transcriptomic sequencing technologies treat tissues as a bulk entity and provide only an average signal from all cells, which obscures the contributions of key pathogenic cell subsets and makes it challenging to capture the core disease drivers. The recent emergence of single-cell RNA sequencing (scRNA-seq) technology has overcome this limitation by resolving cellular composition and transcriptional states at the resolution of individual cells. Meanwhile, Mendelian randomization (MR) analysis uses genetic variants as instrumental variables to infer causal relationships between gene expression and disease risk, thereby mitigating confounding. Therefore, integrating the high resolution profiling of scRNA-seq with the causal inference capability of MR is essential to systematically identify the specific T cell subsets and key regulatory genes commonly dysregulated in BD and IBD, which is crucial for deciphering their shared pathology.

Although existing evidence suggests a potential link between BD and IBD, no study has yet performed an integrated analysis of single-cell and genomic data for both diseases. Therefore, we employed scRNA-seq and Mendelian randomization (MR) analysis to systematically explore the shared molecular mechanisms within specific immune cell subpopulations of BD and IBD. We first analyzed the scRNA-seq data of the peripheral blood of BD and IBD, and identified CD4^+^ TEM_TH1-like cells as a key CD4^+^ T cell subset that significantly decreased in both diseases. Subsequently, we identified differentially expressed genes within this subset by combining expression quantitative trait loci (eQTL) and GWAS datasets. Then MR analysis was applied to determine the relationship between gene expression and disease risk. We further investigated the association between BD and IBD using the experimental autoimmune uveitis (EAU) mouse model, which recapitulates BD-related ocular inflammation. Additionally, the downregulation of BTN3A2 was validated using independent external datasets and blood samples from BD patients with uveitis. Overall, this study identifies BTN3A2 as a shared protective factor in BD and IBD and provides experimental evidence linking BD-associated ocular inflammation to intestinal pathology, offering a new basis for developing common treatment strategies for both diseases.

## Methods

### Data sources

We obtained data for MR and colocalization analyses from the IEU OpenGWAS project, which included outcome data for BD and eQTL information for the BTN3A2 gene (https://gwas.mrcieu.ac.uk/, ebi-a-GCST90018798). ScRNA-seq datasets of peripheral blood mononuclear cells (PBMCs) for BD (GSE198616) and IBD (GSE261334) were downloaded from the GEO database. For external RNA-seq validation, we utilized datasets GSE209567 (BD) and GSE3365 (IBD). For experimental validation, we enrolled 24 patients with active BD who had not been systemically treated with any immunosuppressive therapy, immunomodulatory drugs, or biological agents for at least two weeks prior to blood sample collection, along with 24 gender- and age-matched healthy controls. Among these, samples from 10 patients and 10 controls were used for real-time quantitative PCR (RT-qPCR), 4 from each group for western blot (WB) analysis, and 10 from each group for enzyme-linked immunosorbent assay (ELISA). All included patients with BD fulfilled the international criteria (16).

### Single-cell analysis

We processed the single-cell data using the Seurat package in R platform. First, we performed quality control by retaining cells with 200 to 4000 unique genes and less than 10% mitochondrial gene content. A slightly relaxed threshold (< 10%) was applied for clinical samples from patients with active BD and IBD, as immune cells from inflammatory patients are prone to mild mitochondrial stress during sample processing, and a stringent < 5% threshold would excessively remove valuable effector T cells. Supplementary comparative analysis confirmed that this threshold did not introduce apoptotic or dead cell bias (**Supplementary Figure 1**). The data were then normalized and scaled. Principal component analysis (PCA) was used for dimensionality reduction, and the “limma” and “sva” packages were applied to correct for batch effects. We next clustered the cells using the Louvain algorithm and visualized the results in two dimensions with Uniform Manifold Approximation and Projection (UMAP).

Based on the cellular gene markers provided in the study by Zhang et al. (17), we performed a more detailed subpopulation analysis of T cells. Putative intercellular communication networks were computationally inferred for CD4^+^ TEM_TH1-like cells using the CellChat package. Based on transcriptomic profiles, predicted ligand–receptor interactions were identified to reveal signaling trends and key pathways that may mediate communication between CD4^+^ TEM_TH1-like cells and other cell types. Based on expression profiling, candidate ligand–receptor interactions were predicted, and the strength and direction of these interactions between distinct cell subsets were evaluated. The resulting data were represented using cellular trajectory maps and communication networks, illustrating the intricate signaling networks among cells.

### MR analysis

We identified differentially expressed genes (DEGs) in CD4^+^ TEM_TH1-like cells from the IBD cohort compared to controls. Using the criteria |log2FC| ≥ 1 and an adjusted *P*-value < 0.01, 45 key marker genes were selected for subsequent analysis. These key markers were utilized as exposure variables, with eQTLs acting as genetic instruments, modeling gene expression as a quantitative trait to examine the relationship between genomic variants and transcript levels. We utilized the ebi-a-GCST90018798 dataset as the outcome measure for BD, exploring potential causal relationships between phenotypes within a MR framework. The TwoSampleMR package on the R platform was employed. We mainly utilized the inverse variance weighted (IVW) approach, complemented by MR-Egger regression and the weighted median method, to infer causal effects. Heterogeneity and pleiotropy tests were conducted to evaluate model robustness. By comparing BD patients with healthy controls, the study further analyzed the association between IBD and BD-specific marker genes of CD4^+^ TEM_TH1-like cells. Following MR result visualization, independent MR validation was conducted for the BTN3A2 gene, presenting and evaluating outcomes through forest plots, scatter plots, and funnel plots. Additionally, reverse MR analysis was implemented to enhance conclusion reliability and exclude reverse causality interference. We then used the BIMR and coloc packages in R to perform systematic colocalization of significant genetic loci. Specifically, we conducted colocalization analysis between eQTLs associated with BTN3A2 gene expression and GWAS summary statistics. Regional association plots were generated to visualize the results, effectively depicting the strength of genetic associations under varying conditions and the genomic distribution of significant variants.

### Key gene analysis and experimental verification

We visualized BTN3A2 expression across different cell types using the Seurat package. Subsequently, the BTN3A2 gene was subjected to both KEGG pathway enrichment and communication analyses UMAP visualization illustrated the expression patterns of BTN3A2 across distinct T cell subsets.

### Experimental verification

Peripheral blood samples were collected from BD patients and healthy individuals to verify our findings as follows.

### Human CD4^+^ T cells isolation

Human peripheral blood samples from BD patients and healthy controls were processed to isolate PBMCs through density gradient centrifugation with human lymphocyte separation medium (TBD Science, Tianjin, China), following established protocols and prior reports (18). Subsequently, CD4^+^ T cells were selected from the PBMCs using CD4 microbeads (Miltenyi Biotec, Germany).

### RT-qPCR

Total RNA was extracted from human CD4^+^ T cells using the SteadyPure Universal RNA Extraction Kit. cDNA was subsequently synthesized with the Evo M-MLV RT Kit incorporating gDNA Clean for PCR (Accurate Biotechnology, Changsha, China). Target genes were then amplified via quantitative PCR (qPCR) on a QuantStudio™5 system (Thermo Fisher Scientific) using SYBR Green qPCR Master Mix (MedChemExpress) The primer sequences utilized in the RT-qPCR were listed below: BTN3A2-forward primer: 5’ACAGAGCGGGAAATAAGCCTA3, BTN3A2-reverse primer: 5’GGACGAAGACTCCTCTCCAC3’, β-actin-forward primer: 5’ CCTGGCACCCAGCACAAT3’, β-actin-reverse primer: 5’GGGCCGGACTCGTCATAC3’. Gene expression levels were quantified via the 2^-ΔΔCT^ method, normalizing the data to the endogenous control gene β-actin.

### WB

Proteins extracted from CD4^+^ T cells were separated by SDS-PAGE, transferred to PVDF membranes, and probed with BTN3A2 and β-actin antibodies. After stripping, the membrane was reprobed for β-actin. Bands were visualized and quantified using a Bio-Rad imaging system and Image Lab software.

### ELISA

Serum was separated from the collected peripheral blood samples via centrifugation. The concentrations of BTN3A2 in the serum were then measured using an ELISA kit (AMOY LUNCHANGSHUO BIOTECH, CO., LTD, Fujian, China). Absorbance readings were taken at 450 nm with a Varioskan LUX multimode microplate reader (Thermo Fisher Scientific).

### Animals

Female C57BL/6J mice were obtained from Chongqing Enbi Biotechnology Co., Ltd. and maintained under specific pathogen-free conditions. All experimental procedures were approved by the Ethics Committee of the First Affiliated Hospital of Chongqing Medical University and conducted in accordance with the Association for Research in Vision and Ophthalmology (ARVO) guidelines.

### Induction of EAU and disease scoring

The Mycobacterium tuberculosis strain H37Ra was dissolved in Freund’s adjuvant, and human IRBP651–670 was dissolved in PBS. These were then emulsified together. The EAU model involved subcutaneous injection of 650 μg IRBP and intraperitoneal injection of 1 μg pertussis toxin (Sigma, USA) into the mice. On day 14 post-immunization, clinical evaluations were performed, including slit-lamp photography, fundus imaging, and hematoxylin and eosin (H&E) staining of ocular sections. On day 7 post-immunization, colonic tissues were collected, processed for paraffin sectioning, and stained with H&E and periodic acid-Schiff (PAS) for histopathological analysis. Evaluations were independently and blindly scored by two investigators, as reported previously (19, 20).

### Intestinal permeability assay

Following a 4-hour fasting period, C57BL/6 mice received FITC-dextran (300 mg/kg, MW 4000; MedChemExpress) by oral gavage. Blood was collected from the eyeball 4 hours after administration and centrifuged to isolate serum. Standard curves were generated by serial dilution of FITC-dextran in untreated serum. Fluorescence intensity was measured with excitation at 485 nm and emission at 535 nm.

### Retinal transcriptome sequencing

For RNA-Seq analysis, retinal tissues from EAU and Health group were collected for total RNA extraction. Sequencing was performed by Lianchuan Biotechnology Co., Ltd., and all data were analyzed through its online platform (https://www.omicstudio.cn/tool).

## Results

### Transcriptomic profiles and T cell subset heterogeneity in IBD and BD

**Figure 1:**
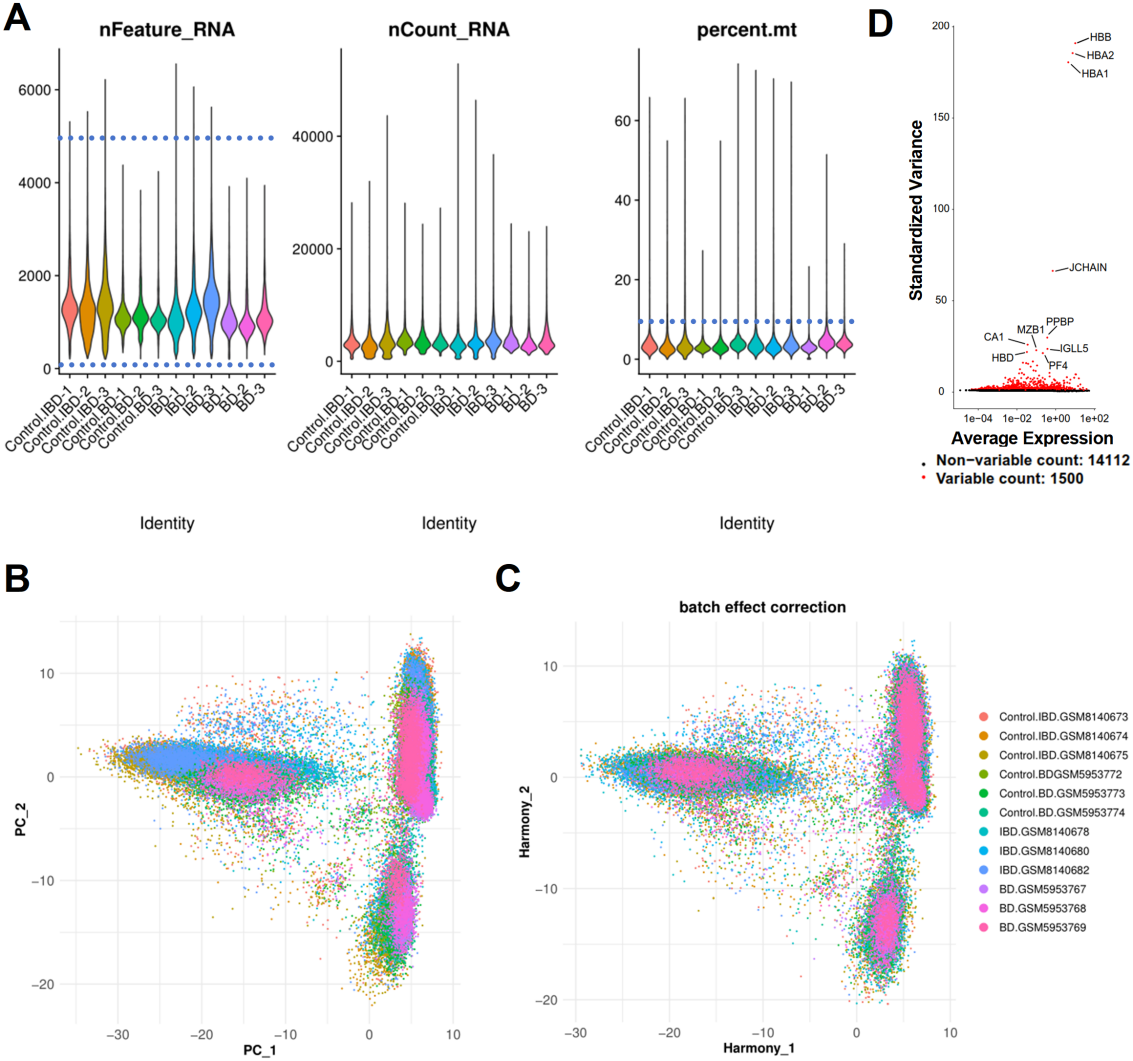
Initial preprocessing and filtering of scRNA-seq data. **(A)** The quality control metrics of sequencing data, number of detected features (nFeature_RNA), the total RNA count (nCount_RNA), The percentage of mitochondrial genes (percent.mt). **(B)** PCA analysis before calibration. **(C)** Scatter plot after batch effect correction. **(D)** Scatter plot of 1500 highly variable genes.

Following quality control to remove low-quality cells and genes, we processed the scRNA-seq data. The expression landscape of each sample is presented in **Figure 1A**. PCA analysis was subsequently applied to extract essential features and reduce the dimensionality of the high-dimensional datasets (**Figure 1B**). The harmony algorithm was used to correct for batch effects, aiming to minimize inter-batch variation in the experimental data. After batch effect correction, samples from different batches showed improved integration in the data space, and we obtained the adjusted UMAP visualization was obtained (**Figure 1C**). Then, 1,500 highly variable genes were identified from 14,112 detected genes through variance analysis.(**Figure 1D**).

**Figure 2:**
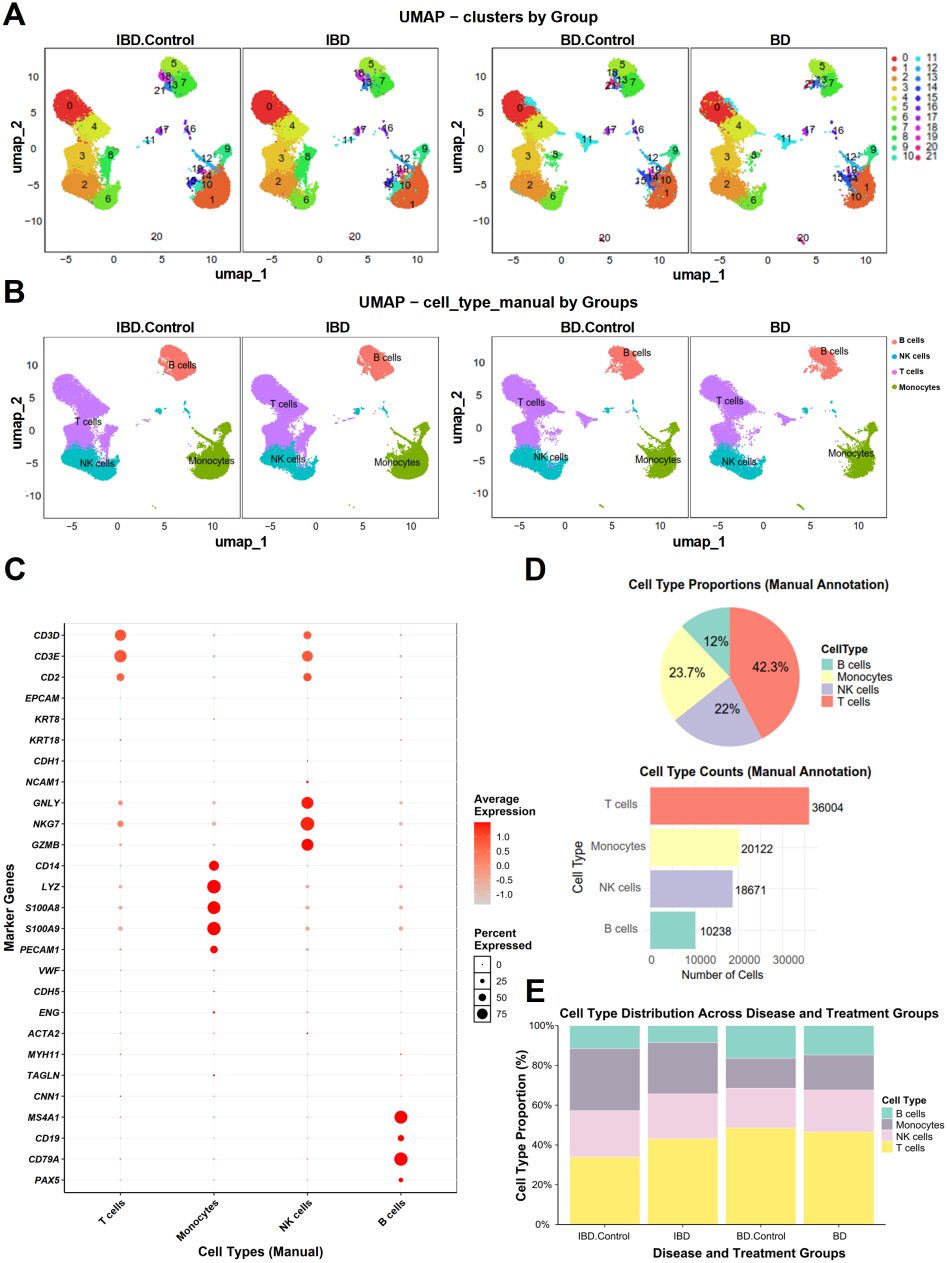
Perform subset analysis on PBMCs. **(A)** UMAP plot revealing the 21 clusters of cells in BD and IBD. **(B)** Marker gene-based cell annotation on UMAP plot (Red: B cells, Green: Monocytes, Blue: NK cells, Purple: T cells). **(C)** Marker gene expression profiles of immune cells. **(D)** Cell type proportions. **(E)** Cell type distributions.

Using UMAP for dimensionality reduction and clustering analysis of data from both BD or IBD patients, we identified 4 distinct lineages from the divided 21 clusters based on marker gene sets in these two diseases: T cells (CD3D, CD3E, TRAC, CD2), NK cells (NCAM1, GNLY, NKG7, GZMB), monocytes (CD14, LYZ, S100A8, S100A9), and B cells (MS4A1, CD19, CD79A, PAX5) (**Figures 2A–C**). We then performed statistical analysis on manually annotated cell subpopulations and found that T lymphocytes exhibited the highest number and proportion (**Figure 2D**) in pool of data from both diseases. Subsequent analysis within disease subgroups revealed the highest T cell proportion in BD and IBD uespectivelly (**Figure 2E**). Therefore, T lymphocytes will serve as the focus for our subsequent analyses.We further validated the absence of dendritic cell (DC) contamination by examining the expression of DC-specific signature genes (CD1C, CLEC9A, LAMP3), which were rarely expressed in the monocyte population, confirming the purity of monocyte annotation (**Supplementary Figure 2**).

**Figure 3:**
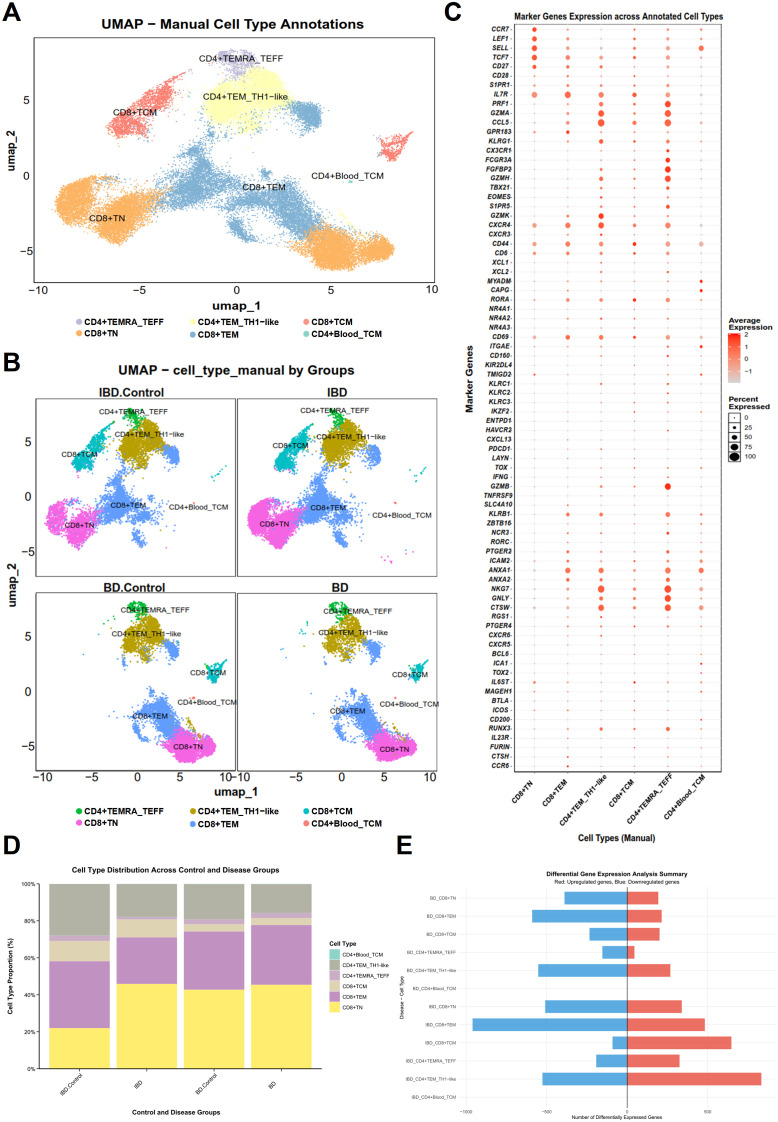
Analysis of T cell subpopulations. **(A)** Distribution of T cell subtypes in UMAP clusters. **(B)** UMAP clusters of T cells subtypes in BD and IBD. **(C)** Marker gene expression profiles defining distinct T cell subsets. CD4⁺ TEM_TH1-like cells were annotated by Th1-associated signature (TBX21, IFNG, CXCR3), while CD4⁺ TEMRA_TEFF cells were defined by terminal effector markers (CD45RA, GZMK, GZMB). **(D)** Proportion of each T cell subset across groups. **(E)** Number of DEGs between control and disease groups for each T cell subtypes.

Following the extraction of T cell subset data, the same procedures for dimensionality reduction, clustering, and batch effect correction were applied. After initial processing, principal components were further analyzed using UMAP for dimensionality reduction, leading to the identification of 14 T cell subsets. These clusters were annotated into six major subsets, namely, CD4^+^ Blood_central memory T cells (CD4^+^ Blood_TCM cells; CCR7^+^ SELL^+^ PTGER2^+^ ICAM2^+^ ANXA1^+^ ANXA2^+^ S1PR1^+^), CD4^+^ effector memory TH1-like cells (CD4^+^ TEM_TH1-like cells; GZMK^+^ GZMA^+^ CCL5^+^ IFNG^+^ RUNX3^+^ EOMES^+^ CXCR3^+^ CXCR4^+^ CD44^+^), CD4^+^ effector memory RA/effector cells (CD4^+^ TEMRA_TEFF cells; KLRG1^+^ CX3CR1^+^ NKG7^+^ PRF1^+^ GNLY^+^ GZMH^+^ TBX21^+^ CTSW^+^ S1PR1^+^ S1PR5^+^), CD8^+^ central memory T cells (CD8^+^ TCM cells; CCR7^+^ SELL^+^ IL7R^+^ CD27^+^ CD28^+^ PRF1^+^ GZMA^+^ CCL5^+^ GPR183^+^ S1PR1^+^), CD8^+^ effector memory T cells (CD8^+^ TEM cells; GZMK^+^ CXCR4^+^ CXCR3^+^ CD44^+^), and CD8^+^ naive T cells (CD8^+^ TN cells; CCR7^+^ LEF1^+^ SELL^+^ TCF7^+^ CD27^+^ CD28^+^ S1PR1^+^) (**Figures 3A, B**). Marker genes defining these subsets are displayed in **Figure 3C**, and the the individual UMAP feature plots of canonical marker genes for T cell subset annotation were presented in **Supplementary Figure 3**. When comparing the proportions of T cell subsets among BD patients, IBD patients, and healthy controls, a similar alteration pattern was observed in both disease groups regarding the CD4^+^ TEM_TH1-like cell population. Specifically, this subset exhibited a decreasing trend in its proportion within the disease groups compared to the healthy controls (**Figure 3D**). **Figure 3E** shows the number of DEGs across various T cell subsets in both disease groups and the control group, and abundant DEGs were found in the CD4^+^ TEM_TH1-like cell population.

It’s worth noting that the two closely distributed effector subsets, CD4⁺ TEM_TH1-like cells and CD4⁺ TEMRA_TEFF cells, were distinguished based on distinct canonical molecular signatures rather than UMAP localization alone. CD4⁺ TEM_TH1-like cells displayed a classic Th1 effector memory phenotype characterized by high expression of TBX21, IFNG, and CXCR3, while CD4⁺ TEMRA_TEFF cells represented a terminal effector subset marked by CD45RA, GZMK, and GZMB expression. Unsupervised Louvain clustering confirmed stable separation between these two subsets, indicating they represent distinct CD4⁺ effector subpopulations rather than a continuous differentiation trajectory or transient activation state. Their close spatial distribution in UMAP reflects their shared functional commitment to effector immune responses, consistent with their strong computationally inferred intercellular communication.

### Intercellular communication analysis

**Figure 4:**
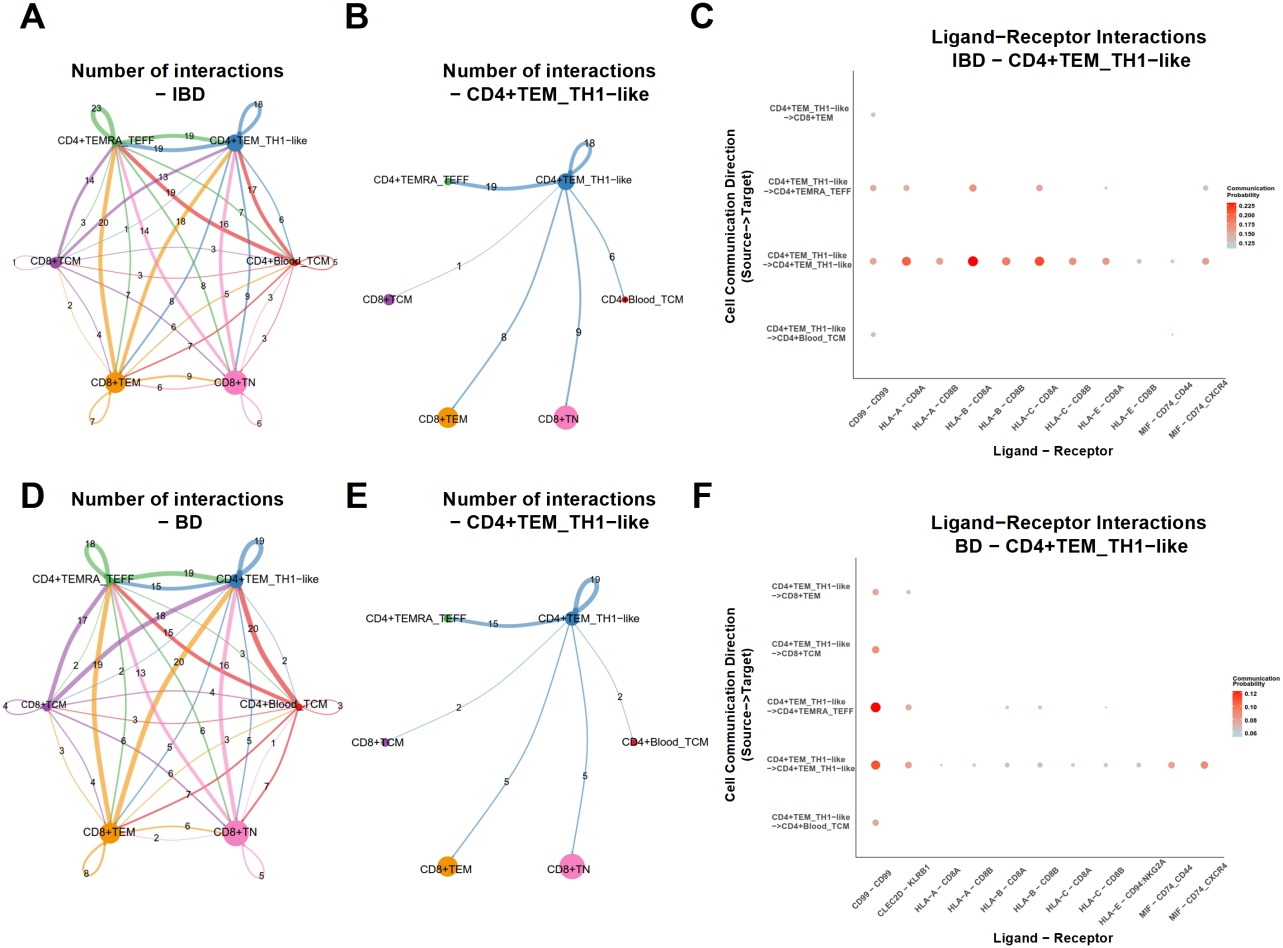
Cell communication analysis for T cell subpopulations. **(A)** The interaction network of CellChat for each subtype of T cell in IBD. **(B)** The interaction network of CellChat for CD4^+^ TEM_TH1-like cells in IBD. **(C)** Dot plot for the enrichment of ligand-receptor pathways for CD4^+^ TEM_TH1-like cells in IBD. **(D)** The interaction network of CellChat for each subtype of T cell in BD. **(E)** The interaction network of CellChat for CD4^+^ TEM_TH1-like cells in BD. **(F)** Dot plot for the enrichment of ligand-receptor pathways for CD4^+^ TEM_TH1-like cells in BD.

To further investigate the mechanisms underlying the numerical and functional alterations in CD4^+^ TEM_TH1-like cells, we employed CellChat to analyze interaction of this subset and other cell subsets. Computational inference revealed prominent putative interactions between CD4^+^ TEM_TH1-like cells and CD4^+^ TEMRA_TEFF cells in both IBD and BD (**Figures 4A, B**). Similarly, predicted interactions between these subsets were also observed in the BD samples (**Figures 4D, E**). We further visualized the ligand–receptor signaling pathways underlying these cellular communications. The interactions between CD4^+^ TEM_TH1-like cells and CD4^+^ TEMRA_TEFF cells were significantly enriched in the HLA-A-CD8A, HLA-B-CD8A, HLA-C-CD8A, and CD99-CD99 pathways in IBD. However, the interactions were primarily enriched in the CD99-CD99 and CLEC2D-KLRB1 pathways in BD (**Figures 4C, F**).

### Key gene screening and MR analysis

**Figure 5:**
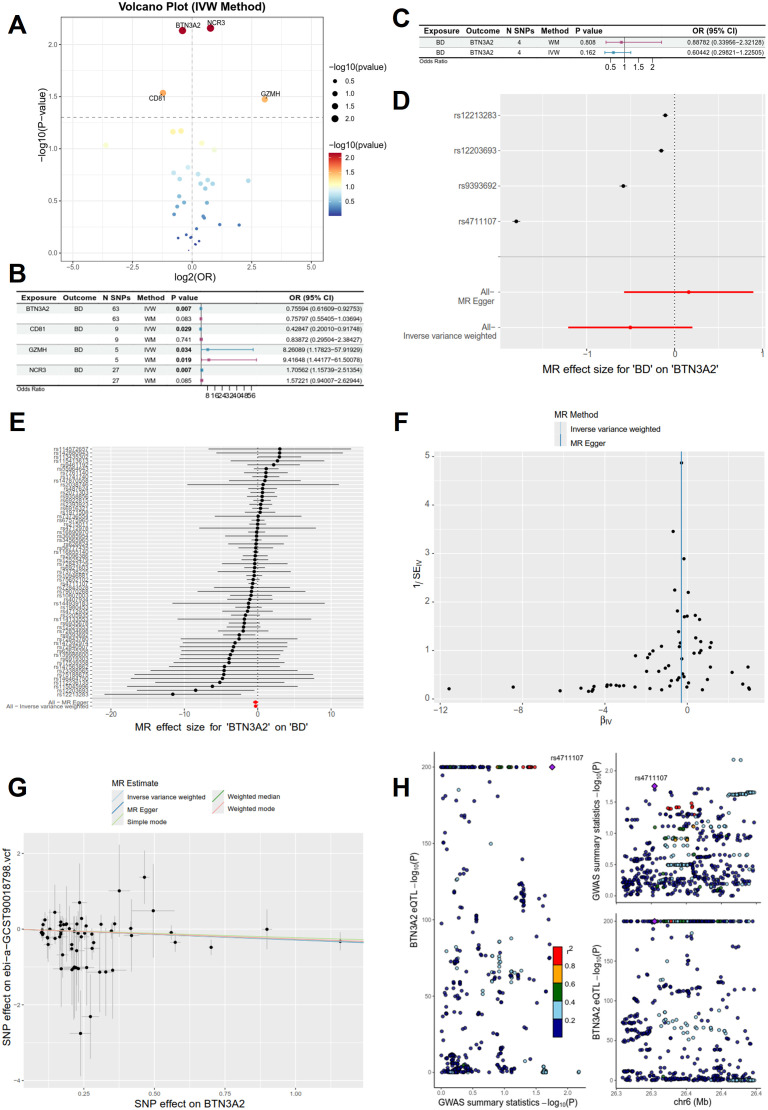
MR and colocalization analyses identify BTN3A2 as a protective factor for BD. **(A)** Visualize significant genes in MR in a volcano plot. **(B)** Forest plot illustrating the results of MR. **(C)** Reverse MR analysis for BTN3A2. **(D)** Forest plot for the reverse MR analysis. **(E)** Forest plot showing the effect sizes of individual SNPs for BTN3A2. **(F)** Funnel plot of SNPs related to BTN3A2. **(G)** The linear regression trend between BTN3A2 and BD risk. **(H)** Colocalization analysis of BTN3A2 eQTLs with BD GWAS data.

To investigate the causal relationship between IBD and BD at the genetic level, we performed a MR analysis. Forty-five key signature genes were identified (|log2FC| ≥ 1 and an adjusted *p*-value < 0.01), through differential expression analysis of the CD4^+^ TEM_TH1-like cell subpopulation in IBD patients. Using these genes as exposures and their associated eQTLs as instrumental variables, we conducted an MR analysis with BD as the outcome. Five MR methods including IVW, MR-Egger regression, simple mode, weighted median, and weighted mode were used to.evaluate causal associations and assess potential pleiotropy. Using a significance threshold of *P* < 0.05 under the IVW method, four genes met the criteria. The effect sizes and significance levels of these genes were visualized using volcano and forest plots (**Figures 5A, B**). NCR3 and CD81 were excluded due to the paradoxical results in MR analysis and differential expression analysis. BTN3A2 and GZMH were selected as key candidates for further investigation. MR-Egger regression and Cochran’s Q test indicated neither significant pleiotropy nor heterogeneity for either BTN3A2 or GZMH.

Results from the IVW method showed that BTN3A2 was associated with a reduced risk of BD (OR: 0.75549, 95% CI: 0.61609–0.92753, *P* = 0.007), while GZMH was associated with an increased risk (OR: 8.26089, 95% CI: 1.17823–57.91929, *P* = 0.034). Reverse MR analysis was also performed for both candidate genes. Using BD as the exposure and GZMH as the outcome, a significant association was observed (OR: 1.57654, 95% CI: 1.20957–2.05483, *P* < 0.001). In contrast, no significant causal effect was found when using BD as the exposure and BTN3A2 as the outcome (*P* = 0.162; **Figures 5C, D**). Therefore, BTN3A2 was selected as a priority target for further investigations. **Figure 5E** displays the effect size of each of the 62 SNPs used as instrumental variables for BTN3A2, with the overall summary estimates located on the left, supporting its role as a protective factor. A funnel plot showed symmetric distribution of the SNPs around the mean effect, indicating no significant directional pleiotropy and supporting the robustness of the results (**Figure 5F**). A scatter plot of the MR associations for BTN3A2 demonstrated a negative correlation between genetically predicted BTN3A2 expression and BD risk, as reflected by the fitted linear trend (**Figure 5G**). This consistent directional effect further supports BTN3A2 as a protective factor for BD. Colocalization analysis revealed spatial concordance between BTN3A2 eQTLs and BD GWAS signals. Notably, the SNP rs4711107, which significantly influences BD risk, was also associated with BTN3A2 expression, suggesting that its protective effect on BD is likely mediated through regulation of BTN3A2 expression (**Figure 5H**).

### Exploration of expression patterns and cellular functions of BTN3A2

**Figure 6:**
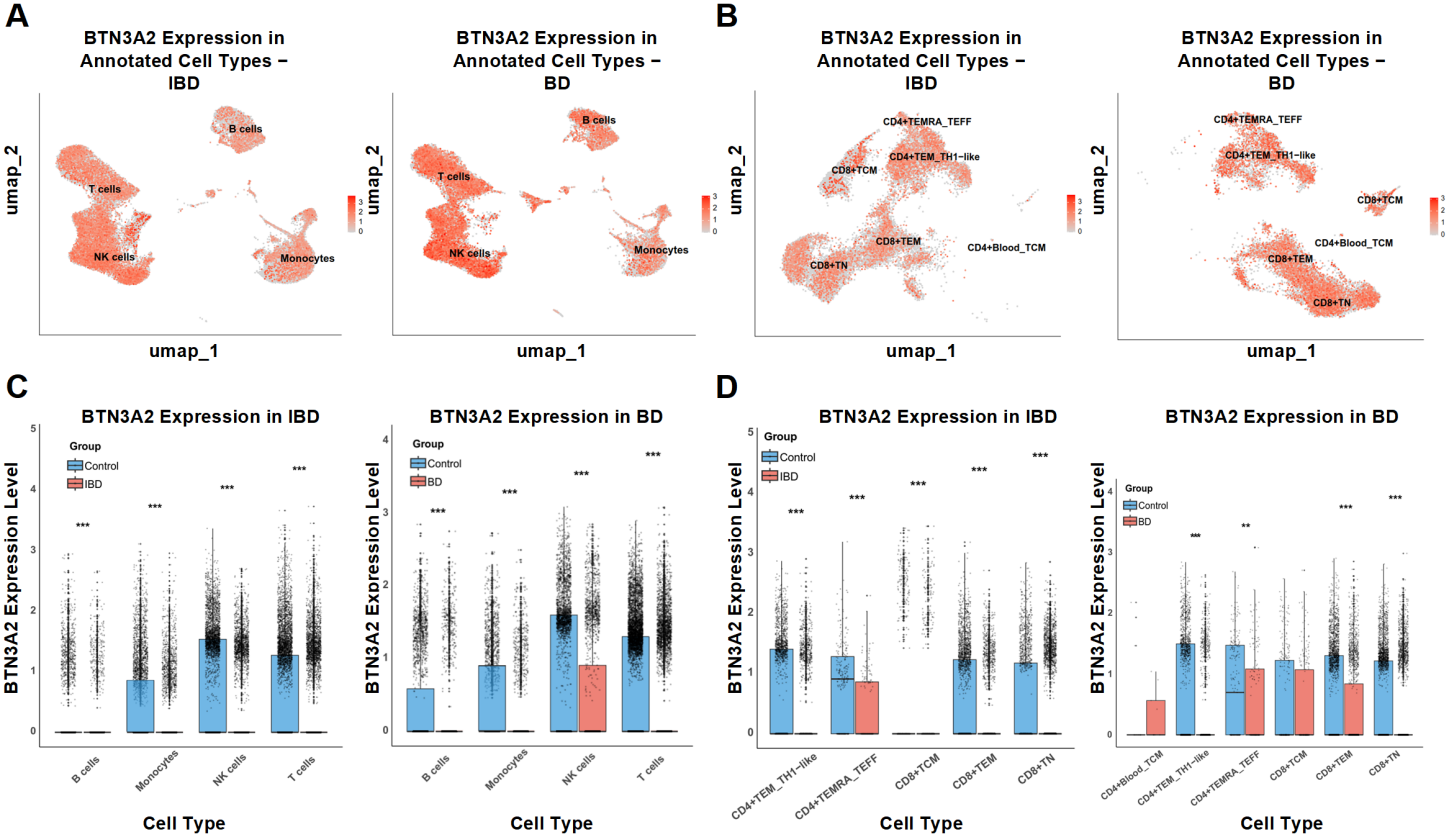
BTN3A2 expression across immune cell subsets in BD and IBD. **(A, B)** UMAP plots showing BTN3A2 expression in PBMC subsets **(A)** and T cell subpopulations **(B)**. **(C, D)** Histograms showing BTN3A2 expression in major immune cell types **(C)** and across T cell subsets **(D)**.

We next investigated the expression pattern of the target gene BTN3A2 in both diseases. **Figure 6** displays UMAP plots illustrating the expression intensity of BTN3A2 across PBMCs subsets (**Figure 6A**) and T cell subpopulations (**Figure 6B**). Statistical results revealed that BTN3A2 expression was significantly downregulated by in B cells, monocytes, NK cells, and T cells in both IBD and BD (all *P* < 0.001) (**Figure 6C**). Among T cell population, BTN3A2 expression was significantly reduced across all six T cell subsets identified in IBD (all *P* < 0.001). In BD, however, a significant downregulation was observed only in the CD4^+^ TEM_TH1-like, CD8^+^ TEM, and CD8^+^ TN cell subsets (all *P* < 0.01) (**Figure 6D**).

**Figure 7:**
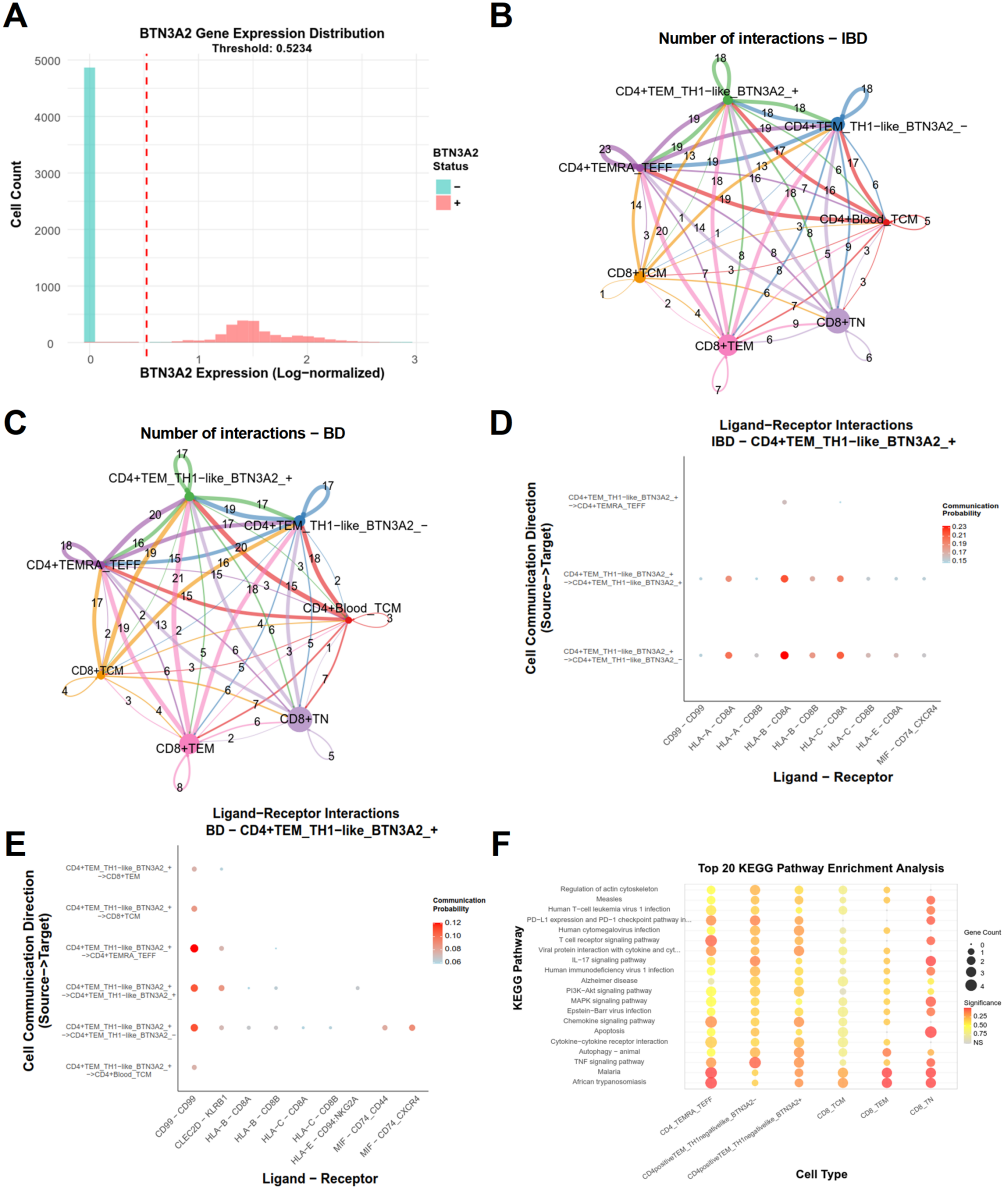
Exploration on the functions of BTN3A2. **(A)** Expression distribution of BTN3A2 in CD4^+^ TEM_TH1-like cells. **(B, C)** Cellular interaction network of BTN3A2^+^ and BTN3A2^-^ CD4^+^ TEM_TH1-like cells in IBD and BD. **(D, E)** Dot plot for the enrichment of ligand-receptor pathways in BTN3A2^+^ CD4^+^ TEM_TH1-like cells in IBD and BD. **(F)** KEGG pathway enrichment analysis for each subtype of T cell.

The decreased expression of BTN3A2 by the CD4^+^ TEM_TH1-like cell subpopulation compared to healthy controls promoted us to investigate functional differences of BTN3A2-positive and BTN3A2-negative subsets in cell communication. A median expression value of 0.5234 was set up as the threshold to classified BTN3A2-positive and BTN3A2-negative subsets (**Figure 7A**). We analyzed and visualized the intercellular communication networks between BTN3A2^+^ and BTN3A2^-^ CD4^+^ TEM_TH1-like cells. The results indicated strong putative communication between BTN3A2^+^ and BTN3A2^-^ CD4^+^ TEM_TH1-like cells, and both of them also exhibited active interactions with CD4⁺ TEMRA_TEFF cells. (**Figures 7B, C**). Further analysis predicted signaling interactions between BTN3A2^+^ CD4^+^ TEM_TH1-like cells and BTN3A2^-^ cells was mediated by the HLA-A-CD8A, HLA-B-CD8A, and HLA-C-CD8A pathways in IBD, whereas, interaction between these two subsets were mainly mediated by the CD99- CD99 and MIF-CD74_CXCR4 pathways in BD (**Figures 7D, E**).

Subsequent KEGG pathway enrichment analysis across T cell subsets showed that BTN3A2^-^ CD4^+^ TEM_TH1-like cells were differentially enriched in pathways such as the TNF signaling pathway, PD-L1 expression and PD-1 checkpoint pathway, and IL-17 signaling pathway. In contrast, BTN3A2^+^ CD4^+^ TEM_TH1-like cells were predominantly enriched in the TNF signaling pathway, Autophagy, and Chemokine signaling pathway (**Figure 7F**).

### EAU exhibit both BD-like ocular inflammation and IBD-like intestinal pathology

**Figure 8:**
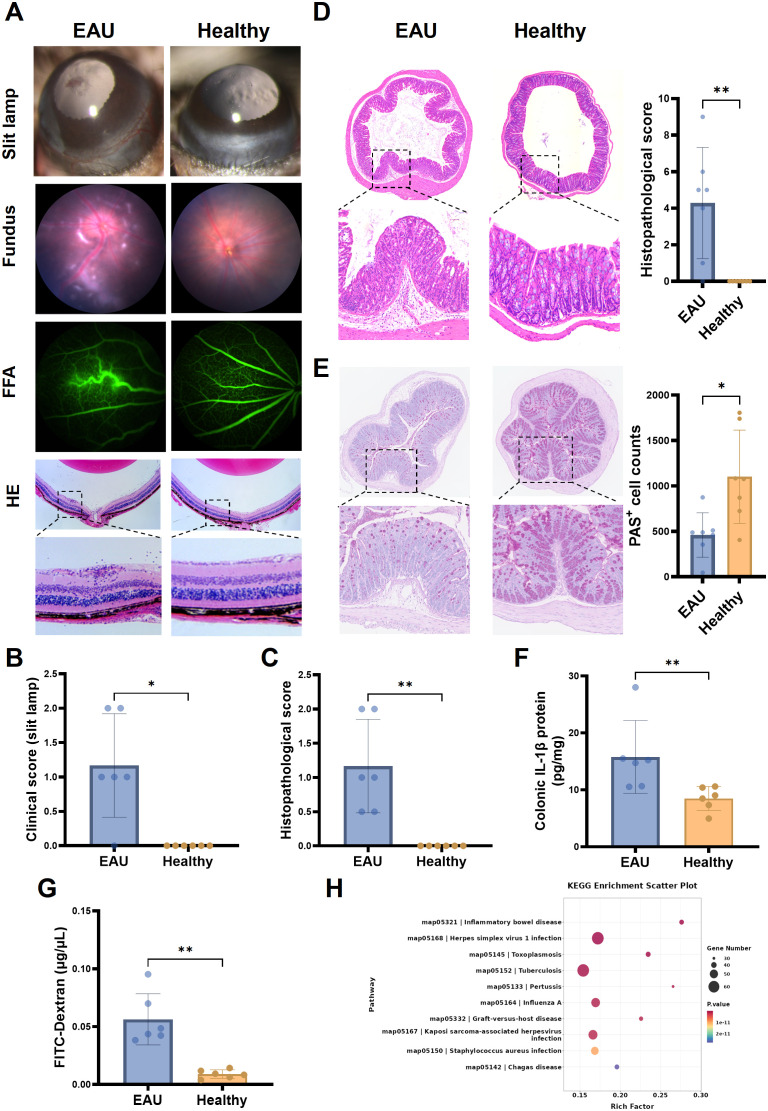
The EAU mouse model recapitulates BD-like ocular inflammation and IBD-like intestinal pathology. **(A)** Representative images of ocular manifestations in EAU mice compared to healthy controls. EAU mice exhibited conjunctival congestion in the anterior segment, retinal vasculitis and soft exudates in fundus photography, and tortuous vessels with vascular leakage in fluorescein angiography **(B, C)** Clinical score and pathological score. **(D–F)** Histological analysis of colonic tissues. H&E staining revealed submucosal edema, inflammatory cell infiltration, and crypt dilation in EAU mice **(D)**, while PAS staining showed goblet cell depletion **(E)**. ELISA demonstrated significantly increased IL-1β protein expression in colonic tissues of EAU mice **(F)**. **(G)** Intestinal permeability assay. EAU mice exhibited significantly increased serum FITC-dextran levels compared with healthy controls, indicating impaired gut barrier integrity. **(H)** KEGG enrichment analysis of retinal transcriptome from EAU mice. Data are presented as Mean ± SD. **p < 0.01, *p < 0.05 versus healthy controls.

To investigate whether BD-associated ocular inflammation could lead to IBD-like intestinal pathology, we employed the EAU mouse model, a well-established system that recapitulates BD-related uveitis. Compared with healthy mice, EAU mice exhibited conjunctival congestion in the anterior segment, as well as typical retinal vasculitis and soft exudates in the fundus. Fluorescein angiography revealed tortuous vessels and vascular leakage (**Figures 8A–C**). Notably, the colons of EAU mice displayed IBD-like pathological changes, including submucosal edema with inflammatory cell infiltration, dilatation of intestinal glands, and goblet cell depletion, accompanied by significantly increased IL-1β protein expression in colonic tissues (**Figures 8D–F**).

To further characterize the intestinal involvement in EAU mice, we evaluated intestinal barrier function. Compared with healthy controls, EAU mice exhibited significantly increased intestinal permeability, indicating that ocular inflammation is accompanied by impaired gut barrier integrity (**Figure 8G**). Furthermore, transcriptomic sequencing of retinal tissues followed by KEGG enrichment analysis identified IBD as the most significantly enriched disease-related pathway (**Figure 8H**). Collectively, these findings demonstrate that the EAU model recapitulates IBD-related pathological features at morphological, molecular, and intestinal barrier functional levels, revealing a shared pathogenic basis between BD and IBD, providing experimental evidence supporting an eye-gut axis.

### BTN3A2 downregulation is validated in independent datasets and BD patients

**Figure 9:**
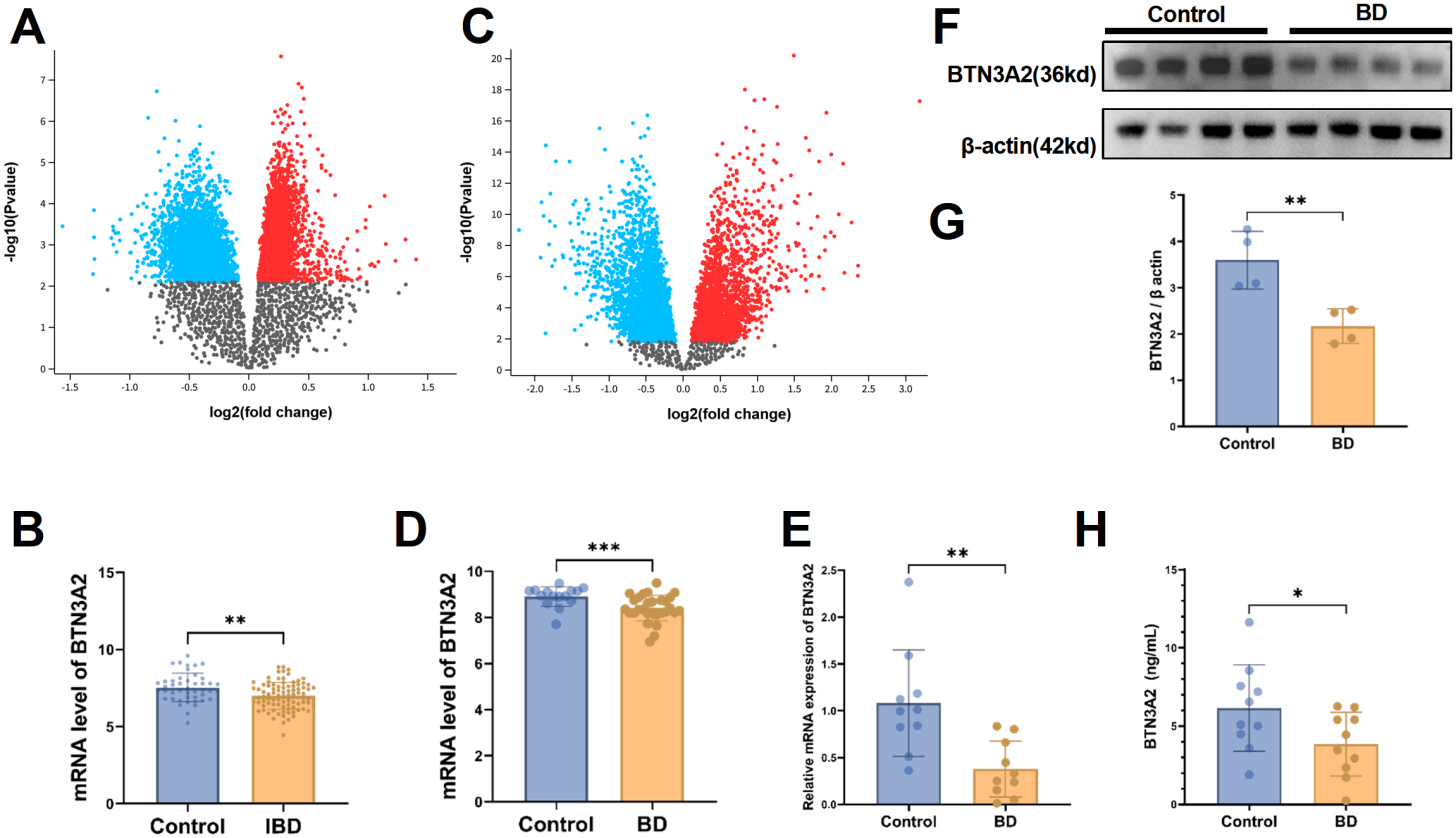
External datasets and laboratory validation. **(A)** The volcano plot displays DEGs between the BD group and control group in the GSE209567 dataset. **(B)** Comparison of the mRNA level of BTN3A2 between the BD group and control group in the GSE209567 dataset. **(C)** The volcano plot displays DEGs between the IBD group and control group in the GSE3365 dataset. **(D)** Comparison of the mRNA level of BTN3A2 between the IBD group and control group in the GSE3365 dataset. **(E)** Results of RT-qPCR for 10 controls vs. 10 BDs. **(F)** Results of WB for 4 controls vs. 4 BDs. **(G)** The statistical graph for WB. **(H)** Results of ELISA for 10 controls vs. 10 BDs. *p < 0.05, **p < 0.01, ***p < 0.001.

We utilized GSE209567 and GSE3365 databases from the GEO as independent validation cohorts. Both datasets contain RNA-seq profiles of PBMCs from disease groups and healthy controls. Volcano plots were used to visualize upregulated (red) and downregulated (blue) genes in BD (**Figure 9A**) and IBD (**Figure 9C**), respectively. Consistent with our findings, BTN3A2 mRNA expression was significantly downregulated in both BD and IBD groups (*P* < 0.001 and *P* < 0.01, respectively, **Figure 9B, D**).

We further performed experimental validation using RT-qPCR, WB, and ELISA on active BD uveitis patients and healthy controls. RT-qPCR showed that BTN3A2 mRNA expression was significantly decreased in CD4^+^ T cells isolated from these patients compared to controls(n=10 per group, *P* < 0.01; **Figure 9E**). Similarly, western blot analysis confirmed a decreased BTN3A2 protein level in CD4⁺ T cells from BD patients (n = 4 per group, *P* < 0.01; **Figures 9F, G**). ELISA of also demonstrated a decresed serum level of BTN3A2 protein in the BD group compared with controls (n = 10 per group, *P* < 0.05; **Figure 9H**).

## Discussion

This study systematically reveals the shared immunopathological target between BD and IBD through an integrated approach combining scRNA-seq, MR, and experimental validation using human samples and animal models. We first identified CD4^+^ TEM_TH1-like cells as the key dysregulated T-cell subset in both diseases, and discovered BTN3A2 as a core protective gene. Further investigation confirmed that BTN3A2 played a crucial role in determining the functional heterogeneity of this T-cell subpopulation. Importantly, our research revealed that BTN3A2 exerts its immunoregulatory effects through different pathways in BD and IBD. Experimental validation utilizing the EAU mouse model has demonstrated that BD-like ocular inflammation is accompanied by IBD-like intestinal pathological changes, thereby providing *in vivo* evidence supporting the comorbidity between these two disorders. Furthermore, independent external datasets and patient samples corroborated the significant downregulation of BTN3A2 at both mRNA and protein levels in BD and IBD patients. These findings offer new perspectives for understanding the common pathogenesis of these two diseases and provide new directions for developing cross-disease therapeutic strategies.

In this study, we first identified CD4^+^ TEM_TH1-like cells as a pathological cellular hub shared by IBD and BD. Single-cell analysis revealed markedly reduced proportions of this cell subset in the peripheral blood of both BD and IBD patients. As an important effector memory T cell population, CD4^+^ TEM_TH1-like cells mediate long-term immune memory and rapid recall responses, and drive Th1-type inflammation through the secretion of cytokines such as IFN-γ and TNF-α. Notably, The phenomenon we observed was not isolated. Previous studies have reported that patients with active BD exhibit significantly lower proportions of peripheral CD4⁺ CD62L^-^ T cells and CD4⁺ effector memory T cells compared to those with inactive BD (18), suggesting that circulating effector T cell reduction may be a common feature during inflammatory flares. The decreased proportion of these cells in peripheral blood likely reflects their active recruitment and accumulation at sites of inflammation, as activated effector T cells are known to migrate from circulation to diseased tissues under chemokine guidance (21–23).

BTN3A2, a member of the butyrophilin family, is an immunoglobulin-like transmembrane protein recognized as an immune checkpoint molecule that plays a crucial role in maintaining self-tolerance (24). It is widely expressed across various immune cells, including T cells, B cells, monocytes, and dendritic cells (25, 26). This study establishes, for the first time, the protective role of BTN3A2 in both BD and IBD through integrative analysis. MR analysis provides strong genetic evidence that BTN3A2 is a shared protective factor for BD, a conclusion robustly supported by the analysis of CD4⁺ TEM_TH1-like cell exposures derived from IBD patients, confirming that this protective effect is shared between the two diseases. Additionally, colocalization analysis further indicated that genetic variants influencing BTN3A2 expression coincide with genomic loci associated with BD risk, suggesting that BTN3A2 links genetic susceptibility to disease manifestation. Although direct mechanistic studies of BTN3A2 in IBD and BD remain limited, evidence from other immune-related diseases provides important clues. MR studies have indicated that elevated BTN3A2 expression in resting T cells is causally associated with a reduced risk of asthma and allergic rhinitis, thereby supporting its anti-inflammatory role (27). Furthermore, BTN3A2 has been demonstrated to safeguard cells against SARS-CoV-2 infection via its interaction with ACE2 (28). To further investigate its mechanism, we stratified CD4^+^ TEM_TH1-like cells into BTN3A2^+^ and BTN3A2^-^ subsets for functional profiling. Pathway enrichment analysis demonstrated that BTN3A2^-^ cells were significantly enriched in classic pro-inflammatory pathways, including TNF and IL-17 signaling. In contrast, BTN3A2^+^ cells were primarily associated with autophagy and chemokine-related pathways, suggesting that BTN3A2 serves as a key regulator of the functional state of this subset. Notably, intercellular communication between these two subsets is mediated by distinct signaling pathways in IBD and BD, accurately reflecting the unique microenvironmental features of the two diseases. These findings offer a molecular-level insight into how shared genetic factors can result in different clinical manifestations.

Multi-dimensional validation at both the animal model and clinical sample levels provided corroborative evidence supporting the protective role of BTN3A2 in BD and IBD. The EAU mice exhibited both BD-like ocular inflammation and IBD-like colonic pathology, which not only recapitulates the clinical features of comorbidity but also suggests that ocular and intestinal manifestations may arise from shared immunopathological mechanisms. Previous studies have documented structural changes in the ileum, including reduced crypt depth and villus length, as well as elevated colonic IL-1β levels in EAU mice (29, 30). Our study further characterized colonic pathological alterations, such as inflammatory cell infiltration, crypt dilation, goblet cell loss, and barrier disruption, thereby expanding the phenotypic spectrum of intestinal involvement in the EAU model. The enrichment of IBD-related pathways in the retinal transcriptome of EAU mice indicated that ocular and intestinal pathologies might be regulated by common molecular pathways. In clinical sample levels, BTN3A2 expression was markedly reduced at both mRNA and protein levels in BD patients with uveitis, and independent external datasets confirmed BTN3A2 mRNA downregulation in both IBD and BD patients, thereby providing clinical relevance for our core findings. Collectively, these complementary lines of evidence from animal models and clinical samples establish a robust foundation for understanding the shared immunopathology of BD and IBD.

This study has several limitations. First, the single-cell sequencing and main MR analyses were both based on peripheral blood samples. Although peripheral blood can largely reflect the systemic immunopathological status of autoimmune diseases and offers high accessibility and convenience for sample collection, this approach cannot directly reflect local conditions in disease target tissues, such as oral ulcers in BD or intestinal mucosa in IBD. The MR analysis was performed using peripheral blood eQTLs rather than tissue-specific eQTLs from the intestine or eye, which are the primary affected sites in BD and IBD. We chose blood eQTLs because they provide the most robust and well-powered genetic instruments for causal inference, and circulating immune cells represent the major pathogenic cell population underlying both diseases. Notably, interrogation of the GTEx database revealed that the cis-eQTL signals of BTN3A2 in blood were highly consistent with those in colonic tissues, supporting the reliability of our causal inference. Nevertheless, with the future availability of large-scale eye-specific and intestinal mucosa–specific eQTL datasets, tissue-stratified MR analysis will help refine the tissue-specific causal roles of BTN3A2 in BD and IBD.

Second, although we identified BTN3A2 as a downregulated shared protective factor in both BD and IBD, and confirmed the co-occurrence of ocular and intestinal inflammation in the EAU model, the present findings are mainly based on expression correlation analysis and observational disease model validation. Direct *in vivo* functional evidence, such as whether genetic or pharmacological modulation of BTN3A2 could causally alter the disease phenotype of the ocular-intestinal comorbidity, remains to be supplemented. Given that BTN3A2 is a primate−specific gene, future studies using non−human primate models are warranted to further elucidate the causal role and underlying molecular mechanism of BTN3A2 in the pathogenesis of this comorbidity, thereby providing more solid experimental evidence for its potential as a therapeutic target.

## Conclusion

In summary, this study identifies CD4^+^ TEM_TH1-like cells as a shared cellular hub in BD and IBD and reveals BTN3A2 as a key protective regulator downregulated in both diseases. Additionally, we provide experimental evidence linking BD-associated ocular inflammation to intestinal pathology. These findings uncover a shared protective target and a pathogenic link between BD and IBD, offering a new basis for developing cross-disease therapeutic strategies.

## Data Availability

The datasets presented in this study can be found in online repositories. The names of the repository/repositories and accession number(s) can be found in the article/**Supplementary Material**.

## Supplementary material

Supplementary Figure 1 Comparison of scRNA-seq data quality between different mitochondrial gene content filtering thresholds. **(A)** Cellular clustering comparison. **(B)** Marker gene expression comparison. **(C)** Core signature gene consistency between datasets filtered with <5% and <10% mitochondrial gene content thresholds.

Supplementary Figure 2 Expression verification of dendritic cell-specific marker genes in PBMC immune cell subsets.

Supplementary Figure 3 Individual UMAP feature plots of canonical marker genes for T cell subset annotation.
