# Supplementary figures and images for: Integrative single-cell transcriptomics and mendelian randomization identifies BTN3A2 as a shared protective factor in Behçet’s disease and inflammatory bowel disease
Source: Front Immunol. 2026 Jun 24;17:1845143. doi: 10.3389/fimmu.2026.1845143 (PMC13341595; doi:10.3389/fimmu.2026.1845143)

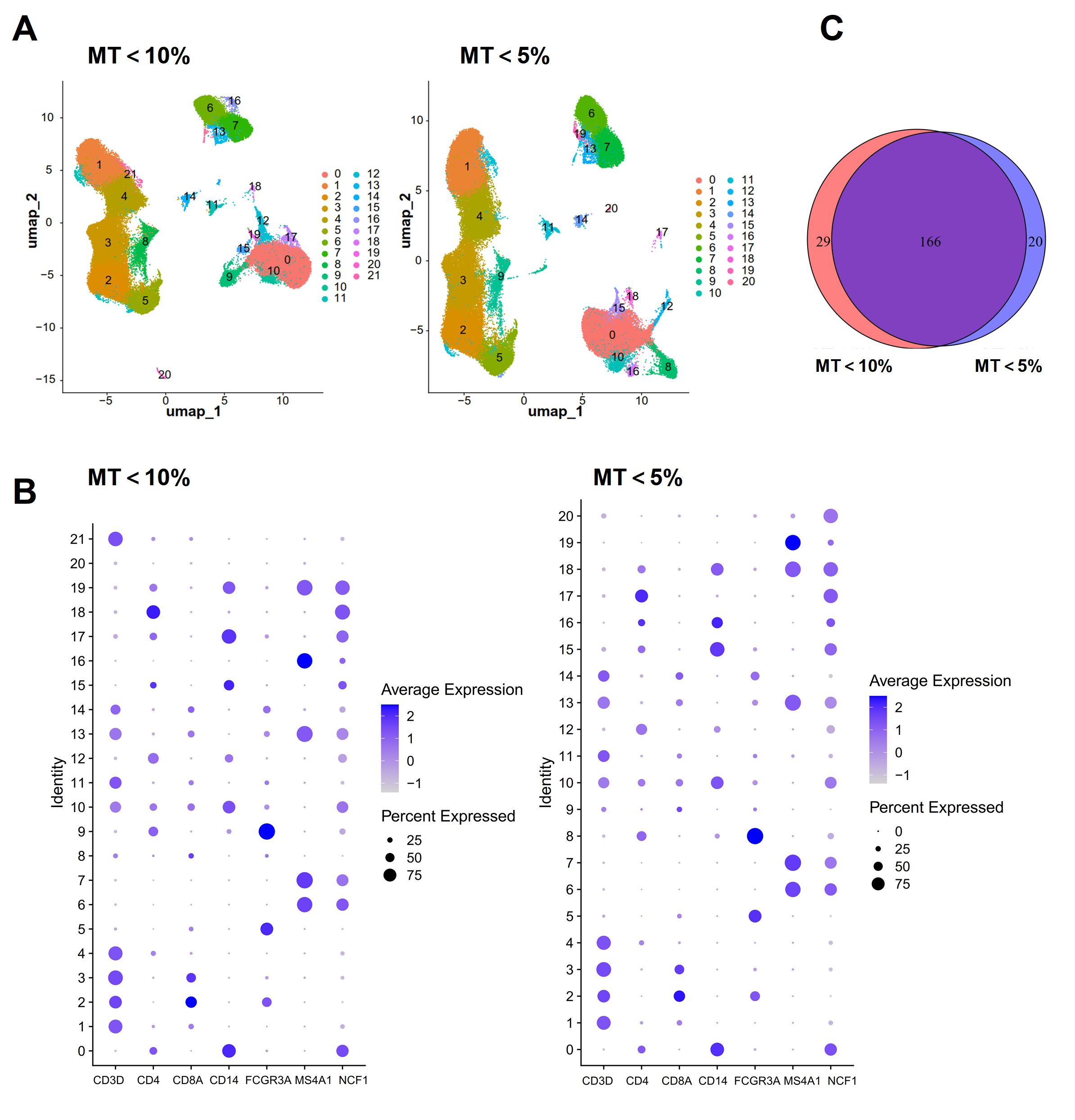

Supplement: Supplementary Figure 1 — Comparison of scRNA-seq data quality between different mitochondrial gene content filtering thresholds. (A) Cellular clustering comparison. (B) Marker gene expression comparison. (C) Core signature gene consistency between datasets filtered with <5% and <10% mitochondrial gene content thresholds. [file Image1.tif]

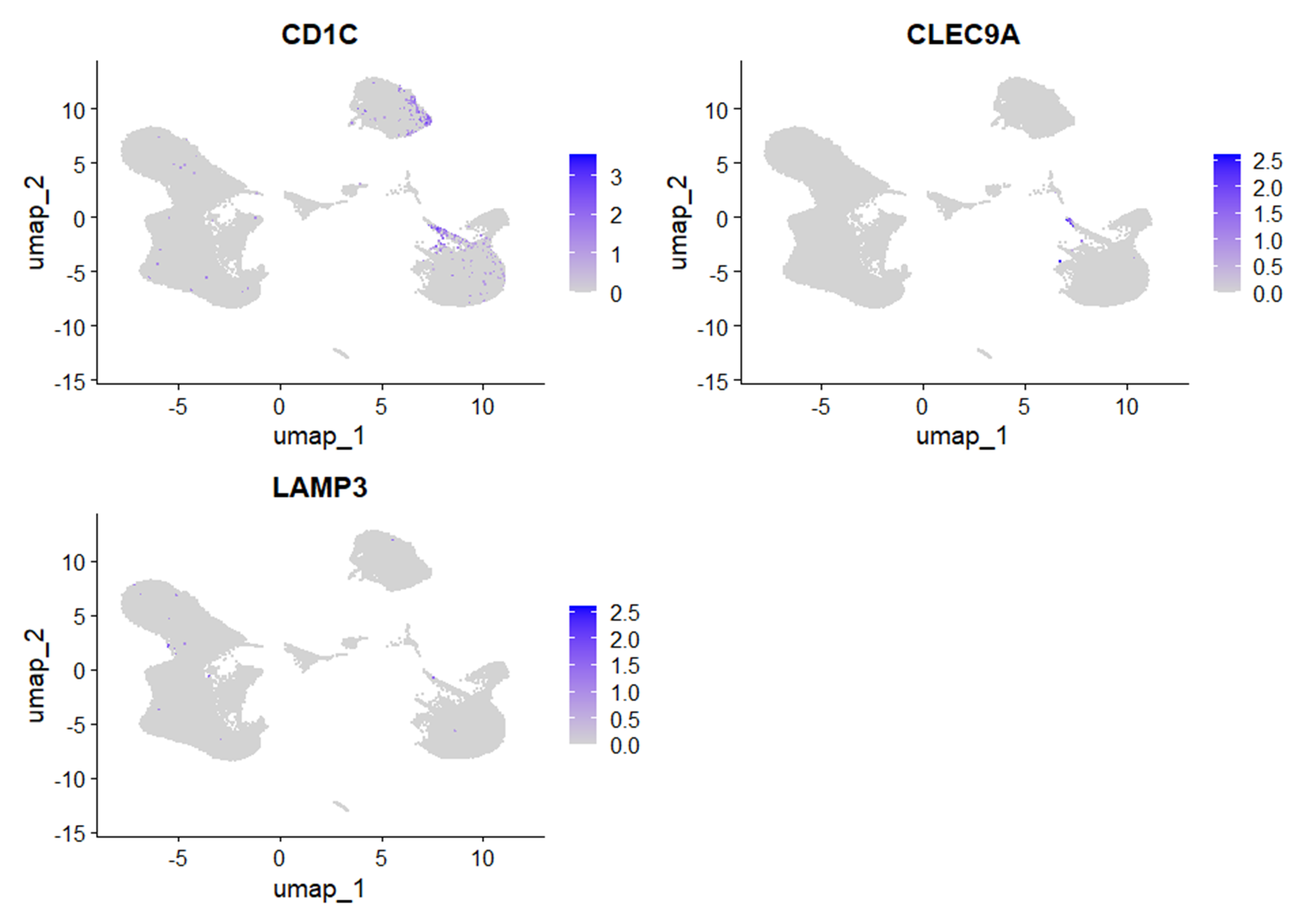

Supplement: Supplementary Figure 2 — Expression verification of dendritic cell-specific marker genes in PBMC immune cell subsets. [file Image2.tif]

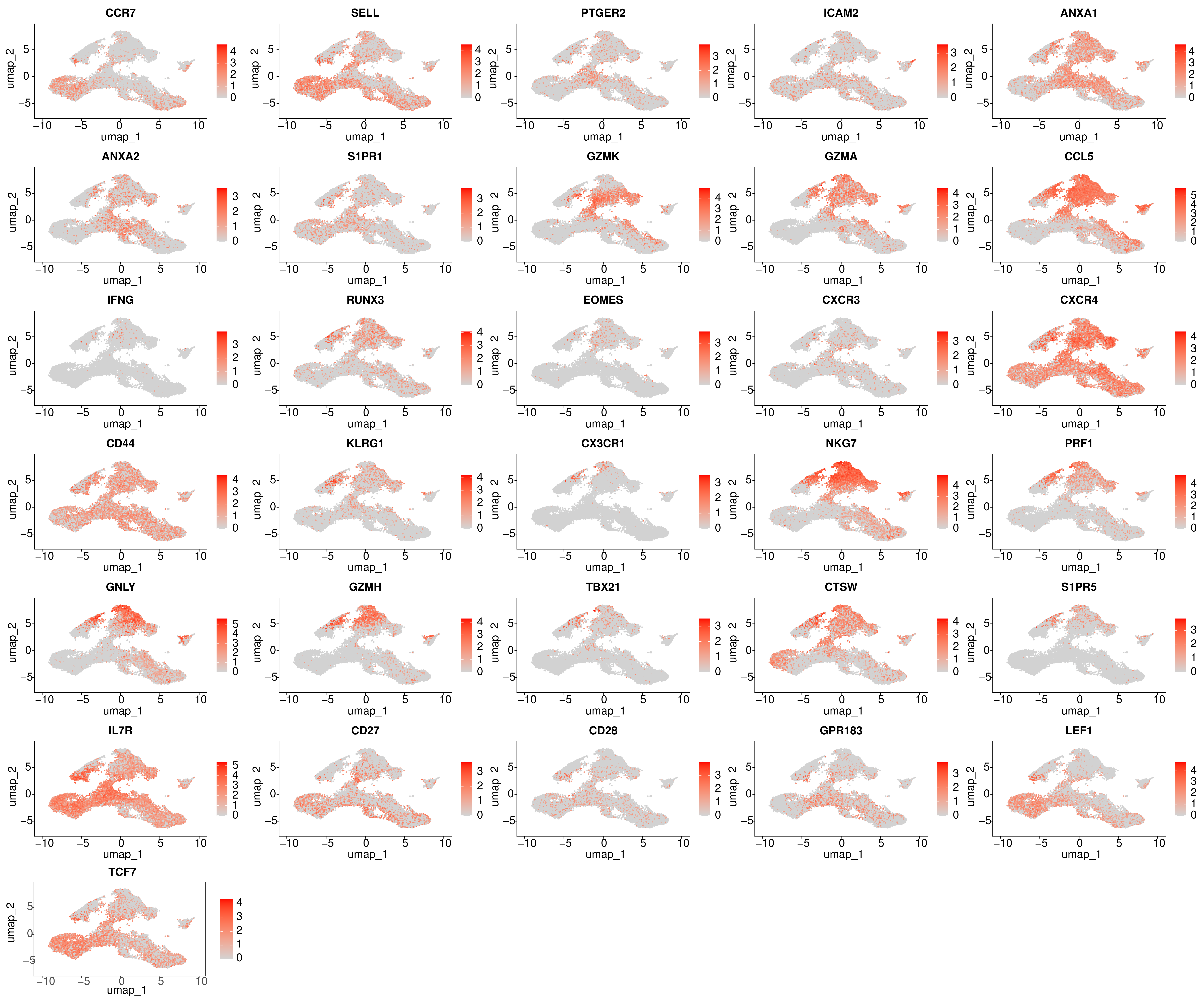

Supplement: Supplementary Figure 3 — Individual UMAP feature plots of canonical marker genes for T cell subset annotation. [file Image3.tif]
